# Physiological Effects of Intermittent Passive Exposure to Hypobaric Hypoxia and Cold in Rats

**DOI:** 10.3389/fphys.2021.673095

**Published:** 2021-05-31

**Authors:** Garoa Santocildes, Ginés Viscor, Teresa Pagès, Sara Ramos-Romero, Josep Lluís Torres, Joan Ramon Torrella

**Affiliations:** ^1^Secció de Fisiologia, Departament de Biologia Cel^.^lular, Fisiologia i Immunologia, Facultat de Biologia, Universitat de Barcelona, Barcelona, Spain; ^2^Departament de Química Biològica, Institut de Química Avançada de Catalunya (IQAC-CSIC), Barcelona, Spain

**Keywords:** cold, erythropoiesis, skeletal muscle, rats, intermittent hypoxia, hypobaria, capillarization

## Abstract

The benefits of intermittent hypobaric hypoxia (IHH) exposure for health and its potential use as a training tool are well-documented. However, since hypobaric hypoxia and cold are environmental factors always strongly associated in the biosphere, additive or synergistic adaptations could have evolved in animals’ genomes. For that reason, the aim of the present study was to investigate body composition and hematological and muscle morphofunctional responses to simultaneous intermittent exposure to hypoxia and cold. Adult male rats were randomly divided into four groups: (1) control, maintained in normoxia at 25°C (CTRL); (2) IHH exposed 4 h/day at 4,500 m (HYPO); (3) intermittent cold exposed 4 h/day at 4°C (COLD); and (4) simultaneously cold and hypoxia exposed (COHY). At the end of 9 and 21 days of exposure, blood was withdrawn and gastrocnemius (GAS) and tibialis anterior muscles, perigonadal and brown adipose tissue, diaphragm, and heart were excised. GAS transversal sections were stained for myofibrillar ATPase and succinate dehydrogenase for fiber typing and for endothelial ATPase to assess capillarization. Hypoxia-inducible factor 1α (HIF-1α), vascular endothelial growth factor (VEGF), and glucose transporter 1 (GLUT1) from GAS samples were semi-quantified by Western blotting. COLD and HYPO underwent physiological adjustments such as higher brown adipose tissue weight and increase in blood-related oxygen transport parameters, while avoiding some negative effects of chronic exposure to cold and hypoxia, such as body weight and muscle mass loss. COHY presented an additive erythropoietic response and was prevented from right ventricle hypertrophy. Intermittent cold exposure induced muscle angiogenesis, and IHH seems to indicate better muscle oxygenation through fiber area reduction.

## Introduction

More than 140 million people worldwide live at high altitude (above 2,500 m) and 40 million people are also exposed to altitude environment due to recreational or other reasons (Moore et al., 1998; West et al., 2012). Altitude is characterized by low barometric pressure and, hence, reduced atmospheric oxygen partial pressure (PO_2_), which in turn results in a decrease in arterial oxygen content leading to tissue hypoxia (Lundby et al., 2009). Mammalian cells are able to sense oxygen changes and to respond to maintain adequate oxygen levels and homeostasis and, thus, ensure cell function and survival (Semenza, 1998). Organisms can react with a wide range of responses to low PO_2_ (Breen et al., 2008; Lundby et al., 2009), but the magnitude of this response to hypoxia is directly related to the dose because the severity of the hypoxemia, the duration of the hypoxic exposure, and its frequency are responsible for the subsequent beneficial or pathological reaction (hormesis) (Navarrete-Opazo and Mitchell, 2014).

Intermittent hypoxia is characterized by long periods of normoxia alternated with periods in hypoxia, resulting in a potentially cumulative effect that begins with the first exposure (Neubauer, 2001; Viscor et al., 2018). During the last few decades, it has been well documented that intermittent hypobaric hypoxia (IHH) could elicit beneficial responses in the organism without detectable adverse consequences. Among other things, it has been shown that IHH enhances erythropoiesis and aerobic capacity, improves muscle capillarization and metabolism, controls hypertension, accelerates tissue repair, ameliorates bronchial asthma, regulates metabolic syndrome, and improves altitude acclimatization (see for review Viscor et al., 2018).

Despite numerous studies focused on the effect of hypobaric hypoxia, very few works have considered the fact that high altitude in natural environments always involves a simultaneous exposure to hypobaric hypoxia and cold stimuli. Exposure to cold is characterized by increased metabolic rate and blood flow in order to respond to the higher oxygen needs by tissues during thermogenesis. However, during hypoxia, cellular oxygen availability is reduced due to a low PO_2_, triggering physiological responses oriented to maintain oxygen supply at the tissue level. Interestingly, during concurrent cold and hypoxia, increased oxygen demands and compromised oxygen availability occur simultaneously (Banchero et al., 1985; Jackson et al., 1987).

Mammals have the ability to respond and adapt to different environmental stressors to guarantee their survival (Breen et al., 2008; Maekawa et al., 2013). During acclimatization to hypoxia, increased ventilation and enhanced oxygen transport and delivery are aimed at improving oxygen extraction and use in cells (Neubauer, 2001). Hypoxia-inducible factor 1α (HIF-1α) is the main mediator of cellular hypoxia, regulating more than 100 genes involved in key processes such as erythropoiesis and iron metabolism, angiogenesis, glucose metabolism, cell proliferation and survival, and apoptosis (Ke and Costa, 2006). When homeotherms are exposed to a cold environment, different behavioral responses, morphological adjustments, and physiological adaptations take place in order to reduce heat loss and increase endogenous heat production and, therefore, maintain a stable core temperature (Deveci et al., 2001b; Deveci and Egginton, 2002; Deveci and Egginton, 2003; Maekawa et al., 2013). The hypothalamic–pituitary–adrenal and hypothalamic–pituitary–thyroid axes play a significant role by means of the upregulation of sympathetic effect over the metabolism of carbohydrates and lipids and increasing the expression of beta-adrenergic receptors in hepatic, adipose, and cardiac cells (Zhang Z. et al., 2018). Shivering thermogenesis, which takes place exclusively in the skeletal muscle, is the primary source of heat in the early phase of cold exposure (Suzuki et al., 1997; Egginton et al., 2001; Deveci and Egginton, 2002; Egginton, 2002; Sepa-Kishi et al., 2017; Mahalingam et al., 2020), while during subsequent cold acclimatization, there is a progressive increase of non-shivering thermogenesis *via* brown adipose tissue (BAT) activation (Suzuki et al., 1997; Deveci and Egginton, 2002; Sepa-Kishi et al., 2017; Mahalingam et al., 2020). Moreover, skeletal muscle is also involved in non-shivering thermogenesis based on the activity of Ca^2+^-ATPase in the sarcoplasmic reticulum (SR) (Sepa-Kishi et al., 2017). Thermogenesis in skeletal muscle is supported by increased oxidative metabolism (Egginton et al., 2001; Egginton, 2002) and mitochondrial plasticity (Mahalingam et al., 2020) and requires adaptive changes to improve the oxygen transport system and, thereby, support oxygen demands (Suzuki et al., 1997; Deveci and Egginton, 2002).

In the 1980s, the group led by Natalio Banchero pioneered several studies investigating the effect of concurrent hypobaric hypoxia and cold in muscle tissue, showing that when cold and hypoxia were applied simultaneously, a greater capillarity was found in the gastrocnemius (GAS) muscle than that observed after acclimation to these two factors separately (Banchero et al., 1985; Jackson et al., 1987). Later on, other researchers studied the possible cross-adaptation between cold and hypoxia on the autonomous nervous system (Lunt et al., 2010). Moreover, we have recently shown that simultaneous exposure to IHH and intermittent cold counteracts the pro-inflammatory effect and the body weight (BW) loss produced by isolated hypobaric hypoxia exposure (Ramos-Romero et al., 2020).

The aim of the present study was to investigate the physiological responses to IHH and intermittent cold when they were applied separately or simultaneously. We used rats as a widespread animal model having in mind that an in-depth study on hematological and muscular responses to these two environmental factors could help to better understand the altitude acclimatization process and find out the usefulness of both stimuli as a non-pharmacological therapeutic treatment for injury recovery or as a tool for training in sport performance.

## Materials and Methods

### Animals

A total of 108 adult male Sprague–Dawley rats (Envigo, Casatenovo, Italy) with an initial weight of 211 ± 28 g (mean ± SD) were used in the study. Animals were housed at 25°C ± 2°C and maintained on a 12-h light–dark cycle, with *ad libitum* access to water and food (even during cold and hypoxia exposures). After 1 week of quarantine, rats were randomly divided into four groups: (1) control (CTRL), maintained in normoxia at 25°C; (2) intermittent cold exposed (COLD); (3) IHH exposed (HYPO); and (4) rats simultaneously submitted to intermittent cold and hypobaric hypoxia (COHY). Animals from these different groups were submitted to the assigned intervention procedure for 9 or 21 days.

All procedures were performed in accordance with European Union guidelines for the care and management of laboratory animals and were under license from the Catalan authorities (reference no. 1899), as approved by the University of Barcelona’s Ethical Committee for Animal Experimentation.

### Intervention Protocols

A hypobaric chamber was used to expose rats to IHH. The hypobaric chamber had 136-L capacity, allowing for two rat cages to be placed inside it. The chamber walls were made of polymethyl methacrylate plastic, allowing for the animals’ behavior to be observed during the protocol. A relative vacuum (low pressure) in the chamber was produced by a rotational vacuum pump (TRIVAC D5E; Leybold-Oerlikon, Köln, Germany) by regulating the airflow rate at the inlet with a micrometric valve. Inner pressure was controlled by two differential pressure sensors (ID 2000; Leybold-Oerlikon, Köln, Germany) connected to a vacuum controller (Combivac IT23; Leybold-Oerlikon, Köln, Germany) driving a diaphragm (DIA) pressure regulator (MR16; Leybold-Oerlikon, Köln, Germany). The target pressure of 577 hPa (equivalent to 4,500 m of altitude) was achieved steadily over ∼15 min. Once this desired level was reached, the internal barometric pressure of the chamber was regulated and maintained by the control system for 4 h. A continuous entry of air through the inlet valve avoided the accumulation of humidity and CO_2_ into the hypobaric chamber. At the end of the session, pressurization to normal barometric pressure was gradually restored over ∼15 min. The intermittent cold exposure (ICE) was performed by introducing the rats into their own cages in a cold room at 4°C. Finally, simultaneous cold and hypobaric hypoxia exposure was carried out by placing the hypobaric chamber in the cold room. All intervention consisted of 4 h/day sessions.

### Blood and Tissue Sampling

Animals from the different groups were sampled the day after the last intervention at two different points in time (9 or 21 days). After being anesthetized, a blood sample was drawn from the abdominal cava vein and analyzed by a hematological analyzer (Coulter Spincell 3; Spinreact, Girona, Spain). Then, both GAS, both tibialis anterior (TA), perigonadal (epididymal) adipose tissue (PAT), interscapular BAT, DIA, and heart were excised from each rat, rinsed in saline solution, and weighed. Skeletal muscles were frozen in precooled isopentane (Sigma-Aldrich) and stored at −80°C until further analysis. At the end, the animals were euthanized by anesthetic overdose.

### Histochemical Procedures

**FIGURE 1:**
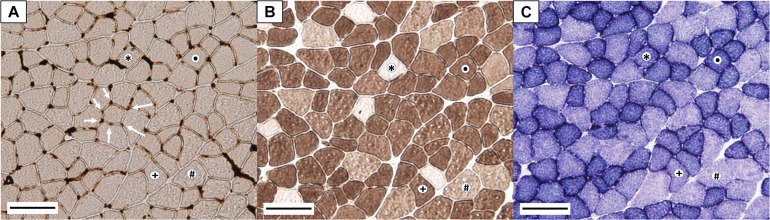
Representative microphotographs of gastrocnemius cross-section histochemical assays. **(A)** Endothelial ATPase stain (arrows indicate muscle capillaries); **(B)** myosin ATPase stain; **(C)** succinate dehydrogenase stain. (^∗^) Slow oxidative fiber (SO); (**⋅**) fast oxidative glycolytic fiber (FOG); (+) fast intermediate glycolytic fiber (FIG); **#** fast glycolytic fiber (FG). Bar represents 100 μm.

Frozen GAS muscles were embedded in a mounting medium (Tissue-Tek; Sakura Finitek Europe, Zoeterwoude, The Netherlands) and cut in serial transverse sections (14–16 μm) using a cryostat (Leica CM3050S, Wetzlar, Germany) at −22°C. Sections were mounted on gelatinized slides (0.02%), incubated for 5 min in a fixing *buffer* (Viscor et al., 1992) in order to prevent shrinkage or wrinkling and finally stained for: (1) myofibrillar adenosine triphosphatase (mATPase), following preincubation in alkaline solution (pH 10.7), to differentiate between slow- and fast-twitch fibers (Brooke and Kaiser, 1970); (2) succinate dehydrogenase (SDH), to identify aerobic and anaerobic fibers (Nachlas et al., 1957); (3) endothelial adenosine triphosphatase (eATPase), to reveal muscle capillaries (Fouces et al., 1993). A representative combined image can be seen in Figure 1. A complete set of representative images for all the histochemical assays from the four experimental groups at the two sampling time points is included as Supplementary Material (Supplementary Figure 1).

### Morphofunctional Measurements

Morphofunctional measurements were performed on microphotographs of stained sections, obtained with a light microscope (BX61; Olympus, Tokyo, Japan) connected to a digital camera (DP70; Olympus, Tokyo, Japan) at × 20 magnification. Since GAS muscle has a heterogeneous muscle fiber type distribution and morphometry, three different zones from each muscle (red, intermediate, and white), as previously described by Armstrong and Phelps (1984), were photographed and further analyzed (Armstrong and Phelps, 1984). All the parameters listed below were measured or calculated from transverse cross-section tissues with an area of 5.5 × 10^5^ μm^2^ using ImageJ software (v. 1.51n; National Institutes of Health, United States).

All muscle fibers were typified according to their metabolic and contractile character (Figures 1B,C) and classified as slow-twitch oxidative (SO), fast-twitch oxidative glycolytic (FOG), fast-twitch glycolytic (FG), or fast-twitch intermediate glycolytic (FIG). SO fibers were unstained for mATPase (pH 10.7) and presented high SDH activity; FOG fibers displayed a dark mATPase and SDH stain; FG fibers presented moderate mATPase stain and remained unstained for SDH assay; and FIG fibers presented moderate to high mATPase and intermediate SDH stain (higher than FG fibers but lower than FOG fibers). Images from eATPase (Figure 1A) were used to measure or calculate the following parameters: fiber cross-sectional area (FCSA), Feret diameter, number of capillaries per 1,000 μm^2^ of FCSA (CCA = NCF⋅10^3^/FCSA), number of capillaries per fiber (NCF), fiber density (FD), capillary density (CD), and capillary-to-fiber ratio (C/F = CD/FD). The mean total number of fibers per rat included for the analysis was 477 ± 112 (±SD). The same researcher performed fiber typing and identification of the capillaries to guarantee that the same criteria were used in all the analyzed images.

### Protein Extraction and Western Blotting

HIF-1α protein expression and two of its target proteins, vascular endothelial growth factor (VEGF) and glucose transporter 1 (GLUT1), were semi-quantified using Western blot technique. Whole GAS muscles were homogenized in urea lysis buffer [6 M urea, 1% sodium dodecyl sulfate (SDS)], supplemented with protease and phosphatase inhibitors (Complete Protease Inhibitor Cocktail and PhosphoSTOP, Sigma-Aldrich), using the Precellys^®^ Evolution tissue homogenizer (Bertin Technologies, Montignt-le-Bretonneux, France). The lysates were centrifuged at 25,000 g for 15 min at 4°C to remove cell debris, and total protein content of the collected supernatants was quantified by the bicinchoninic acid assay (Thermo Fisher Scientific). Muscle protein extracts were solubilized in an electrophoresis loading buffer (62.5 mM Tris⋅HCl, pH 6.8; 2.3% SDS; 10% glycerol; 5% mercaptoethanol; and bromophenol blue), and equal amounts of protein (between 10 and 55 μg) were loaded onto each lane of the gel, and electrophoresis was run on 10% SDS–polyacrylamide gel electrophoresis (PAGE) gels. Ten study samples and one control sample in triplicate (to verify the possible variability between samples across each membrane) were loaded onto each gel. The following internal quality control criteria were used to consider a valid blot: the variation between the control samples had to be less than 20%, and the same control sample was loaded in all the gels to compensate for the variability between gels and to be able to compare them, as described by Martin-Rincon et al. (2019).

After electrophoresis, proteins were transferred to an Immun-Blot polyvinylidene fluoride (PVDF) membrane for protein blotting (Bio-Rad Laboratories). To verify that equal amounts of muscle protein were charged in each well and the efficiency of transference, the membranes were stained with Ponceau S stain (Sigma-Aldrich). For immunoblotting, membranes were blocked with 4% bovine serum albumin (BSA) in TBS-T (Tris-buffered saline containing 0.1% Tween 20) for 1 h at room temperature. To detect our target proteins, membranes were incubated overnight at 4°C with primary antibodies against HIF-1α (MA1-516; Thermo Fisher Scientific), VEGF (MA1-16629; Thermo Fisher Scientific), and GLUT1 (#12939; Cell Signaling Technology) diluted 1:1,000 in 4% BSA-TBS-T. Following this, the membranes were incubated with the corresponding secondary horseradish peroxidase (HRP)-conjugated antibody (#31460 and #31430; Thermo Fisher Scientific) diluted 1:5,000 in 5% Blotto in TBS-T for 1 h at room temperature. The specific bands were visualized with the Clarity TM Western ECL Substrate Kit (Bio-Rad Laboratories), and the chemiluminescence signal was measured using the Odyssey Fc Imaging System (LI-COR Inc. Biotechnology, Lincoln, Nebraska, United States) and quantified with the Image Studio Software (v. 5.2.5, LI-COR Inc. Biotechnology). Muscle signaling data were reported (in arbitrary units) as the sample band intensity relative to Ponceau S stain, and the final data were normalized to the mean value (band densities) of the three control samples loaded on all the gels.

### Statistical Analysis

Data were analyzed using a one-way ANOVA test followed by the Holm–Sidak *post hoc* test or Student’s *t* when appropriate. Statistical comparisons were only tested by comparing groups within the same time point. Statistical significance was set at *p* < 0.05. The results are reported as mean ± standard error (SE) in histograms, tables, and throughout the text, unless otherwise indicated. Most figures are represented through box-and-whisker plots. The box represents the first and third quartiles separated by the median. Whisker end points represent minimum and maximum values, and the mean is represented with a black dot. All statistical tests were performed using the statistical package SigmaPlot 11 (Systat Software, Inc., San Jose, CA, United States, 2008–2009).

## Results

### Animal Body Weight, Time Course, and Body Composition

**FIGURE 2:**
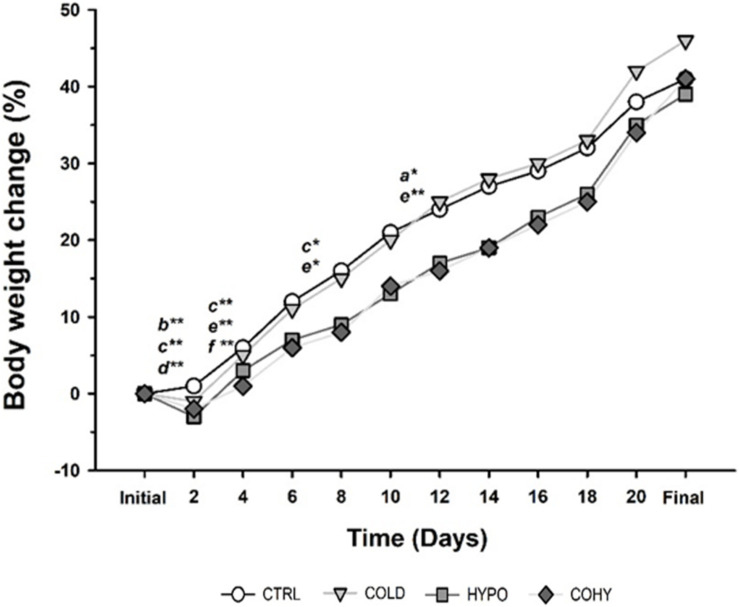
Body weight change during the study. Statistically significant differences are indicated as follows: *p < 0.05, **p < 0.001 between the following pairs: a, CTRL vs. COLD; b, CTRL vs. HYPO; c, CTRL vs. COHY; d, COLD vs. HYPO; e, COLD vs. COHY; f, HYPO vs. COHY. CTRL, control; COLD, intermittent cold; HYPO, intermittent hypoxia; COHY, intermittent cold + hypoxia.

Rats’ BW was monitored throughout the study, and the weight change percentage was calculated every 2 days (Figure 2). The weight of all the animals increased gradually over time, but with some differences between experimental groups at some time points. On day 2 of intervention, animals exposed to IHH (HYPO and COHY) suffered a significant (*p* < 0.001 and *p* < 0.001, respectively) BW loss compared to CTRL, whose BW increased slightly respective to its initial values. Moreover, HYPO presented a significantly reduced BW gain in relation to COLD (*p* < 0.001). Between days 2 and 4 of the study, COHY exhibited significant lower growth than the other groups (*p* < 0.001), while no differences were found across the rest of the experimental groups. On day 8, COHY presented, compared to the previous day, a significantly lower BW increase than CTRL and COLD (*p* = 0.005 and *p* = 0.009, respectively). Nevertheless, from days 10 to 12, only COLD exhibited changes, specifically, larger BW increase than CTRL and COHY (*p* = 0.006 and *p* = 0.001, respectively). No more statistical differences were found among the different experimental groups in the remaining measures. Data showing the percentage changes in BW from the start to the end of the study are shown in Table 1. After 9 days, a statistically significant lower BW gain in animals exposed to hypoxia (alone or combined with cold) when compared to CTRL was evident. COHY also presented reduced BW gain compared to COLD (*p* < 0.026). However, after 21 days, COLD presented a significantly higher weight gain than the rest of the groups.

**TABLE 1:** Rats’ body composition parameters.

	**9 days**				**21 days**			
	**CTRL**	**COLD**	**HYPO**	**COHY**	**CTRL**	**COLD**	**HYPO**	**COHY**
	***n* = 14**	***n* = 15**	***n* = 11**	***n* = 16**	***n* = 14**	***n* = 13**	***n* = 11**	***n* = 14**
BW (Δ%)	24.8 ± 1.2	22.6 ± 1.7	18.7 ± 1.5*	16.5 ± 1.6**^#^	41.7 ± 3.2	48.9 ± 1.9*	37.7 ± 2.6**^##^**	36.1 ± 1.9**^##^**
Muscle (GAS+TA)/ 100 g BW	1.68 ± 0.01	1.64 ± 0.03	1.80 ± 0.09**^##^	1.74 ± 0.03	2.62 ± 0.04	3.08 ± 0.0**	3.35 ± 0.11**^##^	3.01 ± 0.06**^$$^
PAT/BW (mg/g)	8.98 ± 0.41	8.13 ± 0.37	7.81 ± 0.46	8.47 ± 0.62	10.00 ± 0.39	10.07 ± 0.37	8.38 ± 0.38*^#^	10.3 ± 0.43^$^
BAT/BW (mg/g)	1.31 ± 0.08	1.73 ± 0.10**	0.76 ± 0.04**^##^	1.43 ± 0.04**^#$$^**	1.12 ± 0.06	1.38 ± 0.05*	0.89 ± 0.03*^##^	1.33 ± 0.27*^$$^
Diaphragm/BW (mg/g)	1.93 ± 0.05	1.90 ± 0.05	1.80 ± 0.04	1.74 ± 0.13	1.81 ± 0.08	1.82 ± 0.05	1.83 ± 0.09	2.03 ± 0.10
Heart/BW (mg/g)	3.35 ± 0.04	3.34 ± 0.06	3.48 ± 0.10	3.43 ± 0.07	3.37 ± 0.08	3.41 ± 0.10	3.40 ± 0.07	3.59 ± 0.07
Right ventricle/total heart (%)	24.4 ± 0.8	25.0 ± 0.7	28.3 ± 1.4*^#^	24.5 ± 0.5**^$^**	25.6 ± 1.2	26.3 ± 1.2	28.8 ± 0.8	26.4 ± 0.9

Data are presented as mean ± standard error. BW, body weight; GAS, gastrocnemius muscle; TA, tibialis anterior muscle; PAT, perigonadal adipose tissue; BAT, brown adipose tissue. CTRL, control; COLD, intermittent cold; HYPO, intermittent hypoxia; COHY, intermittent cold + hypoxia. Statistically significant differences are indicated as follows: *p < 0.05 vs. CTRL, **p < 0.001 vs. CTRL, ^#^p < 0.05 vs. COLD, ^##^p < 0.001 vs. COLD, ^$^p < 0.05 vs. HYPO, ^$$^p < 0.001 vs. HYPO.

Rats exposed to hypoxia for 9 days exhibited higher limb lean mass (GAS+TA weight) than COLD and CTRL, although these differences were only statistically significant when hypoxia was applied alone (HYPO) (*p* < 0.001 vs. COLD; *p* = 0.006 vs. CTRL). After 21 days, all animals submitted to any intervention sessions (cold and/or hypoxia) showed a significantly higher GAS+TA weight than CTRL (*p* < 0.001). There were also significant differences in lean mass weight between HYPO and COHY (3.35 ± 0.11 g vs. 3.01 ± 0.06 g, *p* = 0.001). Regarding adipose tissue, IHH alone induced a statistically significant decrease in BAT/BW ratio, which was already appreciated after 9 days and was maintained throughout the whole time that the intervention lasted. Furthermore, after 9 days of ICE sessions, COLD presented a significantly higher BAT/BW ratio than CTRL, HYPO, and COHY (*p* < 0.001, *p* < 0.001, and *p* = 0.005, respectively). Nevertheless, after 21 days, COLD group only presented significant differences in BAT weight when compared to CTRL and HYPO (*p* = 0.003 and *p* < 0.001, respectively). Regarding PAT, no statistical differences were found among the different groups on day 9 of the study, but a significant reduction in PAT/BW ratio was found in HYPO compared with the other three groups after 21 days of IHH sessions.

No changes were found in DIA or in heart weight between different experimental groups at any time point (Table 1). Nevertheless, a significant increase in the right ventricular index (calculated as the ratio of right ventricle to whole heart weight) was found in HYPO at day 9 of the experimental period (vs. CTRL, *p* = 0.003; vs. COLD, *p* = 0.012; vs. COHY, *p* = 0.004).

### Hematological Parameters

**FIGURE 3:**
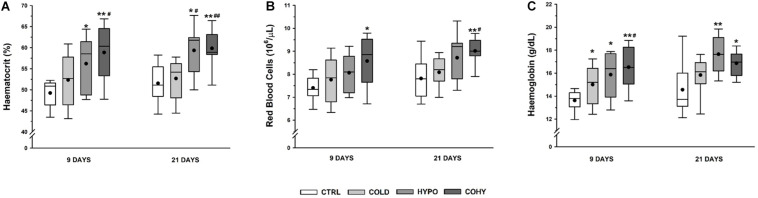
Hematological parameters after 9 and 21 days. **(A)** Hematocrit; **(B)** red blood cell counts; **(C)** hemoglobin concentration. CTRL, control; COLD, intermittent cold; HYPO, intermittent hypoxia; COHY, intermittent cold + hypoxia. Statistically significant differences are indicated as follows: *p < 0.05 vs. CTRL, **p < 0.001 vs. CTRL, ^#^*p* < 0.05 vs. COLD, ^##^*p* < 0.001 vs. COLD. Sample size: *n* = 8–13 per group at each temporal time point.

Oxygen-related transport hematological parameters are shown in Figure 3. After 9 days of IHH exposure, alone (HYPO) or combined with cold (COHY), animals had a significantly higher hemoglobin concentration ([Hb]) and hematocrit (Htc) compared to CTRL. COHY also exhibited a significantly higher [Hb] and Htc than COLD after 9 days ([Hb]: *p* = 0.024, Htc: *p* = 0.007) and significantly higher Htc (*p* = 0.001) after 21 days. COLD compared to CTRL increased [Hb] significantly (*p* = 0.021) within the first 9 days of exposure and had non-significantly higher [Hb] after 21 days. Regarding red blood cell (RBC) count, COHY presented significantly higher values than CTRL both after 9 and 21 days of exposure and higher than COLD after 21 days (*p* = 0.007). An almost significantly greater RBC count (*p* = 0.058) was found in HYPO when compared to CTRL on both days 9 and 21.

Table 2 shows hematimetric indices and white blood cell (WBC) and platelet counts. Statistically significant differences were only found after 9 days in COHY when compared to CTRL and COLD in mean corpuscular volume (MCV) and after 21 days in mean corpuscular hemoglobin concentration (MCHC), all groups having higher values than CTRL. All intervened groups showed higher WBC than CTRL after 9 days, while after 21 days, only HYPO presented significantly higher values than the other groups. Regarding platelets, CTRL and COLD had statistically significant higher counts than HYPO and COHY after 9 days, which was not observed after 21 days.

**TABLE 2:** Rats’ blood haematimetric parameters.

	**9 days**				**21 days**			
	**CTRL**	**COLD**	**HYPO**	**COHY**	**CTRL**	**COLD**	**HYPO**	**COHY**
	***n* = 11**	***n* = 13**	***n* = 8**	***n* = 12**	***n* = 9**	***n* = 12**	***n* = 9**	***n* = 11**
MCV (fL)	66.7 ± 0.7	67.6 ± 0.6	68.6 ± 0.4	70.4 ± 1.1*^#^	65.2 ± 0.9	65.2 ± 0.8	66.6 ± 0.3	66.5 ± 0.6
MHC (pg)	18.4 ± 0.3	18.8 ± 0.6	19.4 ± 0.2	19.6 ± 0.2	18.5 ± 0.5	19.1 ± 0.3	19.22 ± 0.3	18.68 ± 0.2
MCHC (g/dL)	27.7 ± 0.3	28.6 ± 0.3	28.3 ± 0.3	28.1 ± 0.3	27.5 ± 0.1	29.9 ± 0.5*	29.0 ± 0.5*	28.4 ± 0.3**^#^**
WBC (10^9^/L)	7.0 ± 0.5	12.5 ± 0.5*	10.9 ± 1.4*	11.3 ± 1.3*	6.4 ± 1.7	6.9 ± 0.6	10.4 ± 1.7*^#^	6.8 ± 0.6**^$^**
Platelets (10^9^/L)	522 ± 22	603 ± 71	392 ± 34*^#^	406 ± 31**^#^**	497 ± 37	587 ± 44	488 ± 34	451 ± 32

Data are presented as mean ± standard error. MCV, mean corpuscular volume; MHC, mean corpuscular hemoglobin; MCHC, mean corpuscular hemoglobin concentration; WBC, white blood cells. CTRL, control; COLD, intermittent cold; HYPO, intermittent hypoxia; COHY, intermittent cold + hypoxia. Statistically significant differences are indicated as follows: *p < 0.05 vs. CTRL, ^#^p < 0.05 vs. COLD, ^$^p < 0.05 vs. HYPO.

### Fiber Cross-Sectional Area and Feret Diameter

**FIGURE 4:**
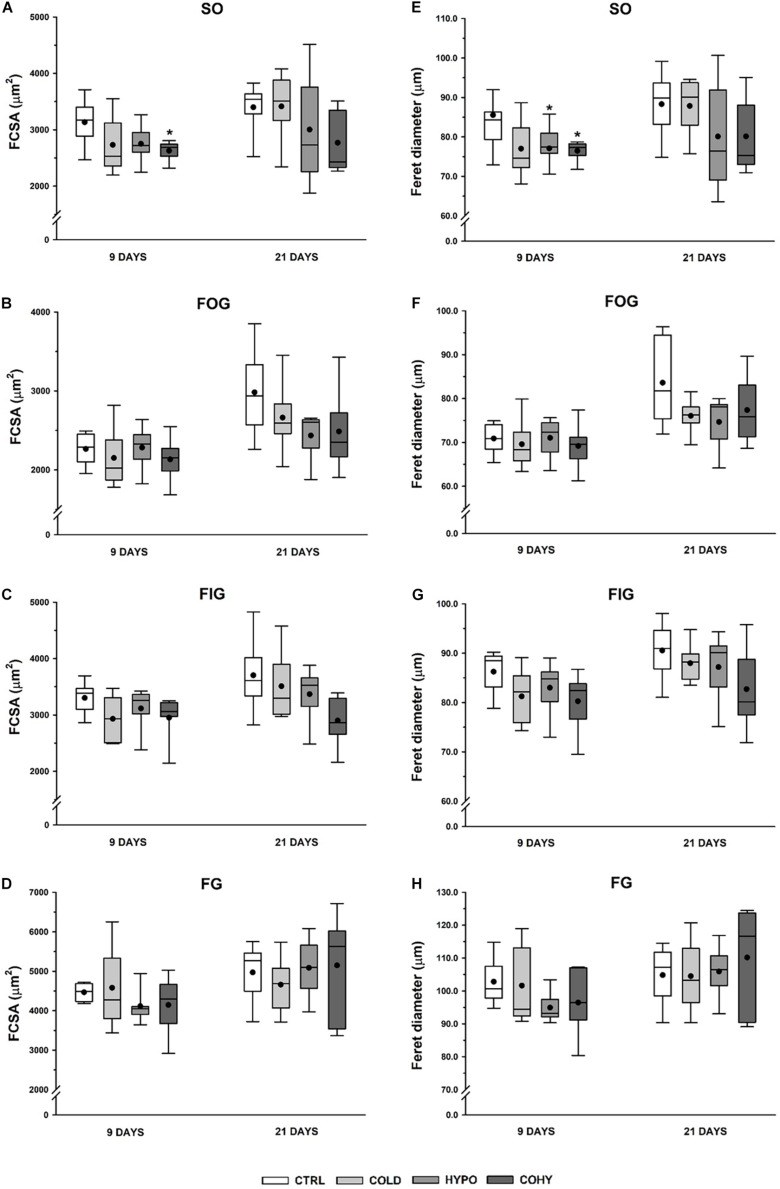
Gastrocnemius muscle fiber morphometry after 9 and 21 days in the different fiber types. **(A–D)** Fiber cross-sectional area (FCSA), **(E–H)** Feret diameter. SO, slow oxidative; FOG, fast oxidative glycolytic; FIG, fast intermediate glycolytic; FG, fast glycolytic. CTRL, control; COLD, intermittent cold; HYPO, intermittent hypoxia; COHY, intermittent cold + hypoxia. Statistically significant differences are indicated as follows: *p < 0.05 vs. CTRL. Sample size: *n* = 6 per group at each temporal time point.

Figures 4A–D show the FCSA measurements of the different fiber types presented separately. All rats subjected to any treatment for 9 days presented a slight FCSA reduction relative to CTRL group in SO fiber type, which is statistically significant (*p* = 0.049) in COHY. After 21 days, SO fibers in COLD-treated animals presented a similar mean FCSA to CTRL (3,314 ± 258 μm^2^ vs. 3,400 ± 225 μm^2^), while both hypoxic groups had a non-significant FCSA reduction (Figure 4A). After 9 days, FOG and FIG fibers (Figures 4B,C) had very similar values in all groups. However, after 21 days, a consistent trend in FOG and FIG fiber size reduction was evident in groups exposed to intermittent hypoxia (HYPO and COHY). These reductions were not statistically significant and ranged from 16 to 18% in FOG fibers and from 10 to 24% in FIG fibers. Finally, no changes were found in FG fibers FCSA among the different groups at any time point.

Feret diameter parameter shows the longest distance between any two points along the section boundary, also known as maximum caliper. As shown in Figures 4E–H, COLD, HYPO, and COHY showed smaller maximum Feret diameter than CTRL in SO fiber type after day 9 (*p* = 0.095, *p* = 0.043, and *p* = 0.007, respectively). After 21 days, only HYPO and COHY presented smaller mean maximum Feret diameter than CTRL, but without reaching significant differences (80.1 ± 6.6 and 80.1 ± 4.5 vs. 88.3 ± 4.0) (Figure 4E). No changes on Feret diameter were found in FOG and FIG fibers at 9 days of intervention protocols (Figures 4F,G). However, at 21 days, a trend toward smaller mean maximum Feret diameter was found in FOG fibers of all treated groups, and smaller mean was observed in FIG fibers of COHY. No changes were found in FG fibers in any group at any temporal point.

### Individual Fiber Capillarization

**FIGURE 5:**
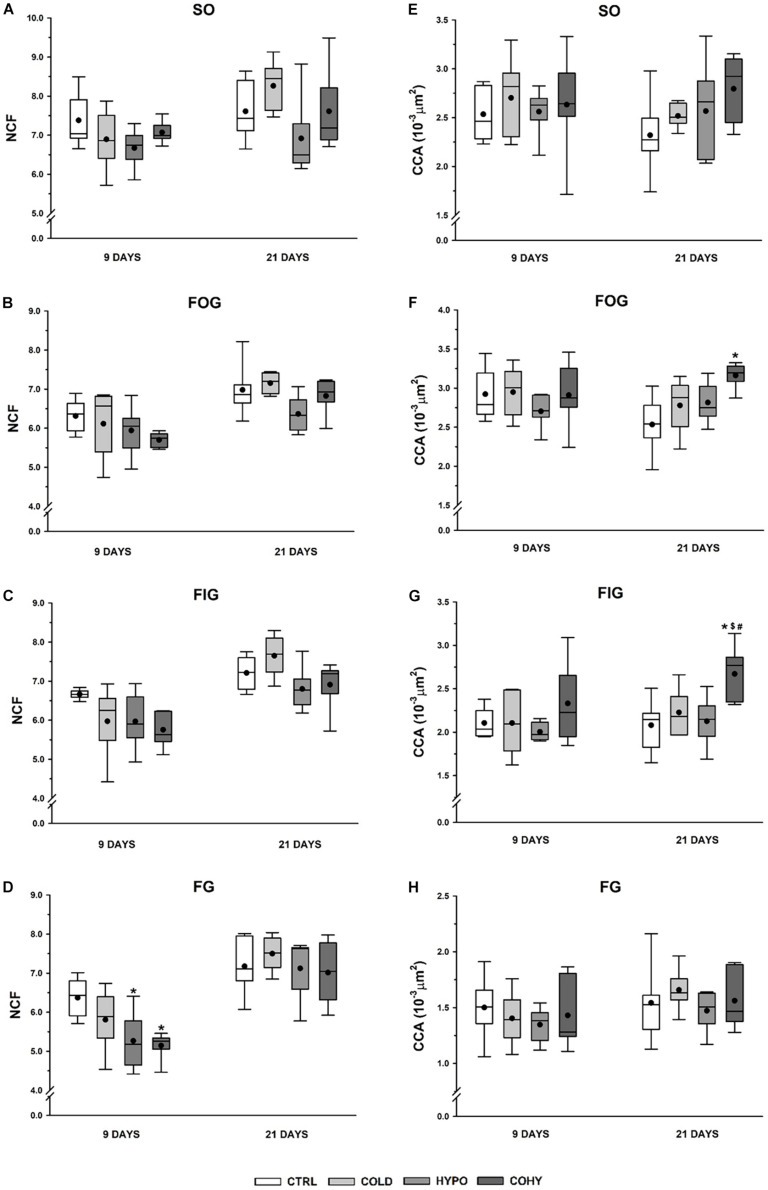
Gastrocnemius individual fiber capillarization after 9 and 21 days in the different fiber types. **(A–D)** Number of capillaries per fiber (NCF), **(E–H)** fiber capillarization index (CCA). SO, slow oxidative; FOG, fast oxidative glycolytic; FIG, fast intermediate glycolytic; FG, fast glycolytic. CTRL, control; COLD, intermittent cold; HYPO, intermittent hypoxia; COHY, intermittent cold + hypoxia. Statistically significant differences are indicated as follows: *p < 0.05 vs. CTRL, ^#^*p* < 0.05 vs. COLD, ^$^*p* < 0.05 vs. HYPO. Sample size: *n* = 6 per group at each temporal time point.

After 9 days, the intervened groups showed in all fiber types a trend to reduce the NCF compared to CTRL (Figures 5A–D). This reduction was statistically significant in FG fibers of the animals exposed to IHH (HYPO and COHY) with a higher significant reduction in COHY (*p* = 0.003) than in HYPO (*p* = 0.022). However, after 21 days, COLD had non-significant increases in the NCF (ranging from 2 to 8%) in all fiber types compared to CTRL. Conversely, HYPO presented a non-significantly lower NCF than CTRL in SO, FOG, and FIG fibers, while COHY did show similar values than CTRL in all fiber types.

The NCF parameter is highly influenced by fiber size. To correct this influence, CCA index was calculated. CCA could be seen as an estimate of the average area to which each adjacent capillary supplies oxygen. After 9 days of any treatment, no changes were found in any fiber type nor between groups for this index (Figures 5E–H), but after the longer exposure period of 21 days, COLD, HYPO, and COHY showed a tendency to increase CCA in SO, FOG, and FIG fiber types. This increase was statistically significant in COHY for FOG and FIG fiber types.

### Fiber Density, Capillary Density, and Capillary-to-Fiber Ratio

**FIGURE 6:**
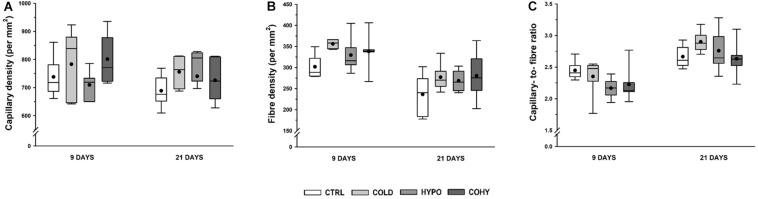
Gastrocnemius global capillarization and morphometric parameters after 9 and 21 days. **(A)** Capillary density, **(B)** fiber density, **(C)** capillary-to-fiber ratio. Sample size: *n* = 6 per group at each temporal time point.

No statistical differences were found in CD between the different groups at any time point, although the COLD group showed a slight increase (6 and 9%) at 9 and 21 days (Figure 6A). Non-significant increases in FD were observed in all intervened groups at both temporal points (Figure 6B). To analyze the relationship between the capillary and fiber densities, the C/F ratio was calculated. At 9 days, the results showed similar values to CTRL for COLD and non-significant reductions (10–13%) for both hypoxic groups (Figure 6C). After 21 days, COLD had 9% higher values than CTRL, while HYPO and COHY remained unaltered.

### Fiber Type Distribution

**FIGURE 7:**
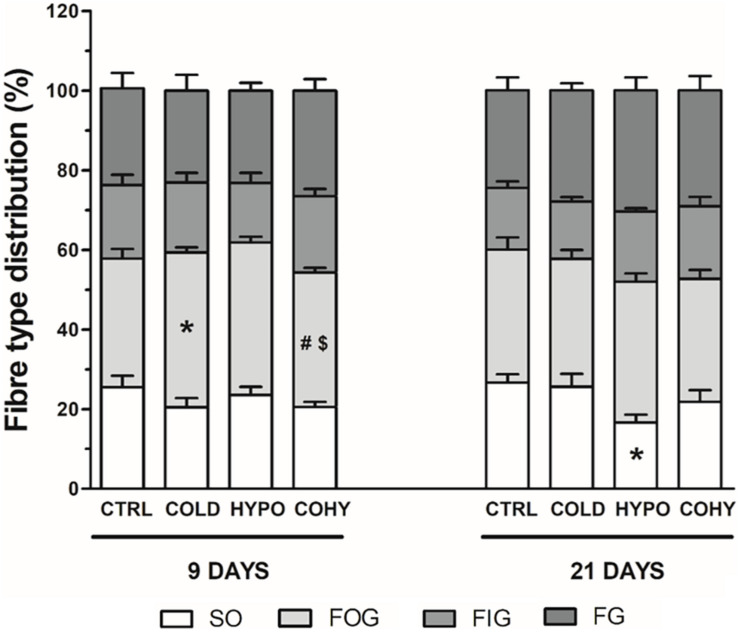
Gastrocnemius fiber type distribution after 9 and 21 days. SO, slow oxidative; FOG, fast oxidative glycolytic; FIG, fast intermediate glycolytic; and FG, fast glycolytic. CTRL, control; COLD, intermittent cold; HYPO, intermittent hypoxia; COHY, intermittent cold + hypoxia. Data are presented as mean ± standard error. *p < 0.05 vs. CTRL, ^#^*p* < 0.05 vs. COLD, ^$^*p* < 0.05 vs. HYPO. Sample size: *n* = 6 per group at each temporal time point.

Fiber type proportions in different groups are shown in Figure 7. After 9 days, the proportion of SO fibers remained unaltered in all conditions studied (averaging 22%), while a significant 10% reduction relative to CTRL was found on day 21 in HYPO. A higher significant percentage of FOG fibers was found in COLD compared to CTRL and COHY and in HYPO compared to COHY after 9 days. Similar percentages of FOG were shown (between 30 and 35%) for all groups after 21 days. FIG and FG fiber proportions remained unchanged in the different groups at both temporal points.

### Hypoxia-Inducible Factor 1α Pathway Protein Expression

**FIGURE 8:**
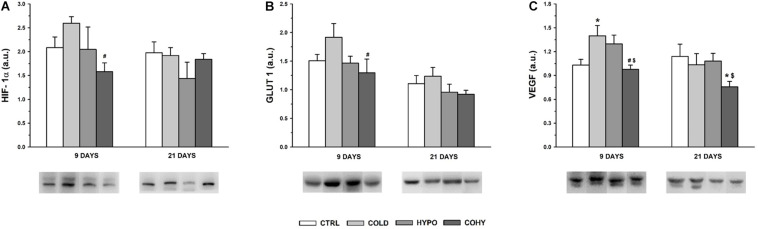
Hypoxia-inducible factor 1α (HIF-1α) signaling pathway protein expression in gastrocnemius muscle after 9 and 21 days. **(A)** HIF-1α; **(B)** glucose transporter 1 (GLUT 1); **(C)** vascular endothelial growth factor (VEGF). CTRL, control; COLD, intermittent cold; HYPO, intermittent hypoxia; COHY, intermittent cold + hypoxia. Data are presented as mean ± standard error and expressed in arbitrary units (a.u.). Statistically significant differences are indicated as follows: *p < 0.05 vs. CTRL, ^#^*p* < 0.05 vs. COLD, ^$^*p* < 0.05 vs. HYPO. Sample size: *n* = 6 per group at each temporal time point.

After 9 days, the expression of the three proteins analyzed had its higher values in COLD (Figure 8). For HIF-1α and GLUT1, the differences were statistically significant compared to COHY and non-significantly higher compared to CTRL (25 and 27%, respectively) (Figures 8A,B), while for VEGF, these were additionally significant with respect to CTRL (Figure 8C). VEGF also had a significantly higher expression in HYPO compared to COHY and a trend to higher values than CTRL (26%). The results obtained for HIF-1α and GLUT1 after 9 days contrasted with the absence of significant differences between groups obtained after 21 days. The significant reduction in VEGF expression in COHY was also evident after 21 days.

## Discussion

### Body Weight and Body Composition Time Course

We have found a significant increment of BW and higher BAT/BW ratio in animals submitted to ICE (COLD) and lower PAT/BW and BAT/BW ratios in animals exposed to IHH alone (HYPO). This adipose tissue reduction could account for the increased skeletal muscle/BW ratio (Table 1). Two more interesting conclusions are derived from our results: (i) simultaneous exposure to intermittent cold and hypoxia (COHY) reverted the decrease of adipose tissue/BW ratios to control values; and (ii) these changes were already evident from 9 days of exposure to the treatment. Although previous studies in non-hibernator rodents have reported that chronic cold exposure either does not produce any changes (Deveci et al., 2001b; Egginton et al., 2001; Deveci and Egginton, 2002, 2003; Sepa-Kishi et al., 2017) or does produce a significant BW reduction (Suzuki et al., 1997; Mizunoya et al., 2014; Tsibul’nikov et al., 2016), our results agree with those reported by others who found that ICE caused an increase in rodents’ BW (Yoo et al., 2014; Tsibul’nikov et al., 2016), which was accompanied by inguinal adipose tissue and BAT weight increase, as well as by the activation of proteins involved in thermogenesis and energy balance, such as uncoupling protein 1 (UCP-1) and proliferator-activated receptor-γ coactivator 1α (PGC-1) (Egginton et al., 2001; Ravussin et al., 2014; Yoo et al., 2014; Tsibul’nikov et al., 2016). These results suggest that intermittent exposure to cold induces adaptive responses preconditioning thermogenic mechanisms for subsequent cold exposures (Yoo et al., 2014; Tsibul’nikov et al., 2016) and did not compromise tissue metabolism and function, maintaining the increase of animal growth and muscle mass (Ravussin et al., 2014; Yoo et al., 2014; Tsibul’nikov et al., 2016).

“Altitude anorexia” has been well documented in high-altitude sojourns. It is characterized by BW loss (muscle mass waste and fat mass reduction), increased metabolic rate, loss of water, and food intake reduction due to a reduced appetite and an increased energy expenditure at altitude (Mathieu-Costello, 2001; Debevec et al., 2014; Dünnwald et al., 2019). The changes of body composition occurring at altitude depend on the severity and the duration of the exposure, as well as the intensity of the physical activity performed. This has been observed in animals housed in both a well-controlled environment (cold, humidity, food availability, activity) and high-altitude expeditions (Kayser et al., 1993; Hoppeler and Vogt, 2001; Mathieu-Costello, 2001; Cabrera-Aguilera et al., 2018; Dünnwald et al., 2019; Flores et al., 2020). Thus, different response patterns have been found depending on the exposure protocols, ranging from the reduction of animals’ weight and retarded animals’ growth (Mathieu-Costello, 2001; Siqués et al., 2006; Cabrera-Aguilera et al., 2018; Flores et al., 2020) to the absence of changes and increase of muscle mass (Deveci et al., 2002; Rizo-Roca et al., 2017, 2018). In concordance with the results of the present study, previous studies carried out in our laboratory based on similar IHH protocols have shown no significant changes in BW (Panisello et al., 2007, 2008; Rizo-Roca et al., 2017, 2018) and in soleus and TA muscle weight (Rizo-Roca et al., 2017) and GAS muscle weight after 3–4 weeks of intermittent hypoxic exposure (Cabrera-Aguilera et al., 2018). Moreover, our results suggest that the lower BW gain observed during the first week of hypoxic stimulus (HYPO) could be directly related to a reduction of fat mass (Mathieu-Costello, 2001). Finally, no reductions in food intake during IHH have been registered in a previous study (Ramos-Romero et al., 2020). Taking all these results together, it seems that 4 h/day of IHH exposure below 5,000 m of altitude and during 9–33 days did not disturb the animals’ growth, preserving muscle mass.

One of the main objectives of this work was to assess the different physiological responses to intermittent cold and hypoxia when they act simultaneously or separately. Regarding registered anatomical measurements, we can observe that COHY presented a reduction in growth-related BW gain during the first days of intervention sessions. Moreover, this reduction was slightly greater than that observed when hypoxia was applied alone (Table 1). However, these differences disappeared in the following days in agreement with previous findings (Banchero et al., 1985), suggesting acclimation of the animals to the stressful environment. Additionally, the high muscle mass suggests that no catabolic process related to physiological stress has occurred. COHY, as well as COLD, presented adaptations to cold exposure by increasing BAT mass.

Another widely known consequence of hypoxia is pulmonary arterial hypertension (PAH) (Ma et al., 2015; Mirrakhimov and Strohl, 2016; Brito et al., 2020), which finally resulted in right ventricle hypertrophy (Ma et al., 2015; Rizo-Roca et al., 2017). This hypoxia-related PAH is produced by pulmonary vasoconstriction and subsequently increased pulmonary vascular resistance and is accompanied by pulmonary vascular remodeling (Pak et al., 2007; Ma et al., 2015; Mirrakhimov and Strohl, 2016; Brito et al., 2020). Our results show a significant increase in right ventricle relative mass (Table 1) after 9 days of intermittent hypoxic exposure, which is in accordance with recent publications (Rizo-Roca et al., 2017). After 21 days, the higher values found in the right ventricle/BW ratio in HYPO were not significant compared to CTRL, which could indicate acclimatization to hypoxic environment as was already suggested by Panisello et al. (2007). Interestingly, the absence of right ventricular hypertrophy when cold and hypoxia stressful stimuli acted simultaneously (COHY, Table 1) suggests a protection against hypoxia-related PAH (Banchero et al., 1985). The mechanism for this phenomenon can be the generalized peripheral constriction induced by cold (Granberg, 1991) that causes a higher vascular resistance in peripheral vessels, thus balancing both vascular resistance and cardiac work in the pulmonary and systemic circuits.

### Hematological Parameters

Our results indicate that intermittent exposure to cold (COLD) induces a moderate increase in blood oxygen transport-related parameters such as Hct, RBC, and [Hb] (Figure 3) and in the hematimetric indices (Table 2), this increase being statistically significant in [Hb] after 9 days and in MCHC after 21 days. The high interquartile variation shown by the box plots depicted in Figure 2, especially after 9 days, could explain why the increase in some parameters failed to show statistical significance. These results match the current knowledge on prolonged exposures to cold, where non-shivering thermogenesis and higher metabolic heat production lead to enhanced oxygen consumption and, hence, the need to increase oxygen-carrying capacity (Deveci et al., 2001b; Maekawa et al., 2013). The results also agree with the studies performed in chronic exposure to cold, where increases of [Hb], RBC, and Htc in non-hibernator rodents were reported (Deveci et al., 2001b; Maekawa et al., 2013).

Regarding intermittent exposure to hypoxia (HYPO), a more consistent, statistically significant and greater response was evident. The hematological responses already took place after the shorter exposure period (9 days) and lasted while the hypoxic stimulus was present at least until day 21 (Figure 2). These results agree with previous studies where similar intermittent exposure protocols were applied (Casas et al., 2000; Esteva et al., 2009; Núñez-Espinosa et al., 2014, 2015). It has been widely known that exposure to high altitude *per se* produces an increase of oxygen transport capacity both in humans and animals except for those adapted to life at high altitude (Vinegar and Hillyard, 1972; León-Velarde et al., 1993; Heinicke et al., 2003; Zhang H. et al., 2018; Laguë et al., 2020). Moreover, intermittently exposed subjects can achieve [Hb], RBC, and Htc values close to those observed in high-altitude residents or in chronically exposed subjects (Heinicke et al., 2003). In fact, it has been previously reported that single exposure to altitude during 90 min is enough to produce a significant increase of EPO in humans (Rodríguez et al., 2000) and that nine alternate sessions of 90 min at 4,000–5,000 m are enough to induce significant hematological adaptive responses to hypoxia (Casas et al., 2000).

Interestingly, we have found that the greatest erythropoietic response appeared when intermittent cold and hypoxia were simultaneously applied (COHY), with significant differences not only to CTRL but also to COLD and greater non-significant values than HYPO, indicating a synergistic effect of cold and hypoxia on erythropoiesis. These results were in contrast to those obtained by Lechner et al. (1981) who reported reduced erythropoietic response after exposure to cold and hypoxia compared to hypoxia alone.

### Fiber Morphometry and Muscle Oxidative Capacity

#### Intermittent Cold Exposure

In the current study, we have seen that ICE produced a tendency toward smaller FCSA, as it happens with chronic exposure (Suzuki et al., 1997). This trend to lower FCSA has been observed even after a short period of ICE (9 days) and was accompanied by light reduction of fiber Feret diameter, which indicates that muscle fibers became smaller and more rounded to reduce oxygen diffusion distance and their capillary domain area, facilitating homogeneous oxygen delivery to whole fiber (Suzuki et al., 1997; Egginton et al., 2001). Regarding individual fiber capillarization, our results show that despite the observed trend to lower NCF compared to CTRL after 9 days of ICE, there were no changes in CCA, suggesting that muscle fiber oxygenation was maintained due to fiber size reduction in the first days of ICE (Suzuki et al., 1997; Egginton et al., 2001; Deveci and Egginton, 2003). However, after 21 days of cold exposure, we found a slightly higher NCF, which in turn was translated into higher CCA, which would lead to an improvement in oxygen delivery per fiber area (Suzuki et al., 1997; Deveci and Egginton, 2002). In the same way, regarding whole-muscle capillarization, a higher C/F ratio was observed at day 21 in COLD, indicating that an angiogenic process occurs after ICE. To ensure adequate oxygen supply, an increase in vascularity is needed (Jackson et al., 1987), but capillary bed is not only important to supply oxygen and fuel to the fibers, it is also relevant to remove metabolic end products and to maintain local thermal balance (Deveci and Egginton, 2002). These observed increases in NCF and in C/F ratio after 21 days of ICE could be directly related to the increase of VEGF protein expression observed in day 9 samples. These results match the hypothesis that angiogenesis is a progressive event in rats (Deveci and Egginton, 2003). Thus, the observed increase in VEGF expression on the first days of ICE underwent histological evidence after 21 days. Additionally, we observed an overexpression of GLUT1 protein in COLD after 9 days of ICE, in accordance with previous findings that suggested that cold adaptation facilitates glucose uptake by skeletal muscle and upregulates oxidative pathway by an insulin-independent manner (Bukowieckh, 1989; Vallerand et al., 1990; Sepa-Kishi et al., 2017). Taken together, all these results indicate that ICE could upregulate HIF-1α signaling pathway based on the higher HIF-1α protein expression and the consequent overexpression of its targets VEGF and GLUT1.

#### Intermittent Hypoxia Exposure

In the present study, we have seen that IHH applied alone (HYPO) produced a trend to smaller FCSA in SO and FG fibers after 9 days, this reduction being accompanied by smaller Feret diameter. After 21 days, the oxidative fibers (SO, FOG, and FIG) largely respond to the hypoxic stimulus, presenting a clear trend to decrease their FCSA and Feret diameter. On the other hand, fibers from HYPO showed the lowest NCF. Considering that NCF and fiber size are closely related, this finding is not surprising because HYPO had smaller fibers overall. It is interesting to note that after 21 days, SO and FOG fibers presented a clear trend to higher CCA probably mostly due to the reduced FCSA found in these fibers. These changes in SO and FOG fiber morphometry indicate that the hypoxic stimulus reduced capillary domain area and diffusion distance in fibers with a predominant oxidative metabolism. It has been shown that hypoxia *per se* is not able to produce angiogenesis in the skeletal muscle, exercise or activity being necessary to activate the angiogenic process (Jackson et al., 1987; Deveci et al., 2001a; Deveci and Egginton, 2002; Rizo-Roca et al., 2018). Deveci et al. (2001a) conducted some experiments in which chronic normobaric hypoxia was applied during different time periods. They found that after 3 weeks of hypoxia, only the active muscles such as DIA (respiratory muscle) and soleus (postural muscle) showed an increased capillarity, while the locomotor muscles such as extensor digitorum longus (EDL) and TA did not show any increase in C/F ratio. Moreover, they did not report changes in FCSA in any of the muscles studied (Deveci et al., 2001a). Nevertheless, after a longer period of hypoxia (6 weeks), fiber capillarity increased in all studied muscles (DIA, SOL, EDL, and TA), and no changes or increases in FCSA were reported (Deveci et al., 2002), confirming the importance of hypoxic dose (exposure time) and the activity in the muscle responses. Thus, our results confirm that IHH at rest did not produce angiogenesis, which may be due to the lack of adequate metabolic or mechanical stimuli necessary to show capillary growth (Deveci et al., 2001a, 2002; Rizo-Roca et al., 2018). Analyzing HIF-1α signaling pathway modulation in HYPO, VEGF protein presented a tendency toward higher expression than CTRL. Despite the higher VEGF expression, we did not find an increase in C/F ratio nor in NCF after IHH at any time point, suggesting that new capillaries there were not generated. Capillary bed arrangement is the result of a combination of continuous and simultaneous angiogenesis, remodeling, and pruning processes in response to hemodynamic and metabolic stimuli (Pries and Secomb, 2014). Therefore, it is important to underline that one role of VEGF is to protect and preserve the capillary bed, even when there is muscle mass loss (Breen et al., 2008). VEGF could be modulated by the HIF-1α signaling pathway (when PO_2_ is low), but also it could be regulated by inflammatory cytokines, high oxidative stress, and energy balance-related AMP-activated protein kinase (AMPK) (Breen et al., 2008). From our results, we are not able to differentiate or identify which stimulus has induced higher VEGF expression after IHH, since HIF-1α pathway upregulation was not clear (Figure 8). Interestingly, the local and systemic adaptations carried out during hypoxic adaptation could attenuate the degree of hypoxia in some tissue cells (Lundby et al., 2009), and maybe our stimulus was not intense enough at muscle to induce angiogenesis. Moreover, HIF-1α is only slightly altered during hypoxic stimulus due to a preexisting high level of this protein in skeletal muscle (Lundby et al., 2009).

#### Simultaneous Intermittent Cold and Hypoxia Exposure

Similarly to what was observed in HYPO, simultaneous exposure to intermittent cold and hypoxia (COHY) showed a reduction of FCSA and Feret diameter in SO fibers within the first days of exposure (9 days). These reductions also affected FOG and FIG fibers, indicating that fibers having the more oxidative profile had a greater response to IHH and ICE, confirming the differential behavior of each fiber type when facing the stressful environmental stimuli (Banchero et al., 1985; Deveci et al., 2002; Panisello et al., 2007; Rizo-Roca et al., 2017, 2018). It is noteworthy that FCSA reductions were larger in COHY than in HYPO and COLD. NCF was reduced in all fibers within the first 9 days, but the absence of changes in CCA indicates that fiber irrigation was maintained. However, after 21 days, COHY presented similar NCF as CTRL, but CCA was significantly increased, reducing the capillary domain in oxidative fibers (SO, FOG, FIG) through fiber area reductions. In contrast to what Banchero et al. (1985) observed after chronic exposure to concurrent cold and hypoxia, intermittent COHY acclimation did not produce increases in CD nor in C/F ratio in any of the time points studied. Moreover, the VEGF angiogenic factor appeared significantly reduced compared to HYPO and COLD, and HIF-1α signaling pathway remained inactivated after COHY exposure (Figure 8). As well as during IHH exposure alone, other systemic and local adaptations, such as increased RBC and [Hb], could attenuate skeletal muscle hypoxia and, hence, would have reduced subsequent muscle adaptive responses.

### Fiber Type Distribution

We found an increase of FOG fiber type proportion in HYPO and COLD after 9 days. In the case of HYPO, this increase was at the expense of reducing FIG proportion, which would be explained by the metabolic flexibility of these fibers to shift to a more oxidative or a more glycolytic character. Surprisingly, increased proportion of FOG percentage in COLD was accompanied by reduced SO fiber type proportion. After 21 days, however, only HYPO presented changes in fiber type composition, showing reduced SO fiber type percentage. This increase of FOG fiber type proportion in HYPO has been previously well documented and could be the cause of the observed reduction in SO percentage due to an inhibition of the well-known type shift from FOG to SO fiber type that takes place during growth in normoxic rats (Ishihara et al., 2000). Moreover, a higher FG fiber type proportion in HYPO and COHY is also observed (22 and 17%, respectively), which would be a more efficient path to produce energy when oxygen availability is compromised (Jackson et al., 1987).

## Conclusion

In conclusion, ICE produced adaptive changes in the physiology of the animals to afford thermogenesis, increasing BAT mass, muscle capillarity, and glucose transport capacity. IHH elicited a reduction in FCSA to enhance blood oxygen transport and preserve muscle fiber oxygenation. Concurrent intermittent cold and hypoxia induced increased BAT, RBC count, and [Hb] and reduced right ventricle hypertrophy but, in contrast to our initial hypothesis, did not produce angiogenesis in skeletal muscle, although reduced capillary supply domain area to facilitate oxygen delivery to the muscle fibers.

## Data Availability Statement

The raw data supporting the conclusions of this article will be made available by the authors, without undue reservation.

## Ethics Statement

The animal study was reviewed and approved by the University of Barcelona’s Ethical Committee for Animal Experimentation.

## Author Contributions

GV, TP and JRT conceived the study. GS and JRT obtained the data. GS, GV, TP, SRR, JLT, and JRT participated in data treatment and discussed the results. GS prepared the manuscript draft. JRT and GV edited the manuscript. All authors approved the submitted version of the document.

## Conflict of Interest

The authors declare that the research was conducted in the absence of any commercial or financial relationships that could be construed as a potential conflict of interest.
