# Supplementary material for: Physiological Effects of Intermittent Passive Exposure to Hypobaric Hypoxia and Cold in Rats
Source: Front Physiol. 2021 May 31;12:673095. doi: 10.3389/fphys.2021.673095 (PMC8201611; doi:10.3389/fphys.2021.673095)
Supplement: Supplementary file 1 [file Image_1.PDF]

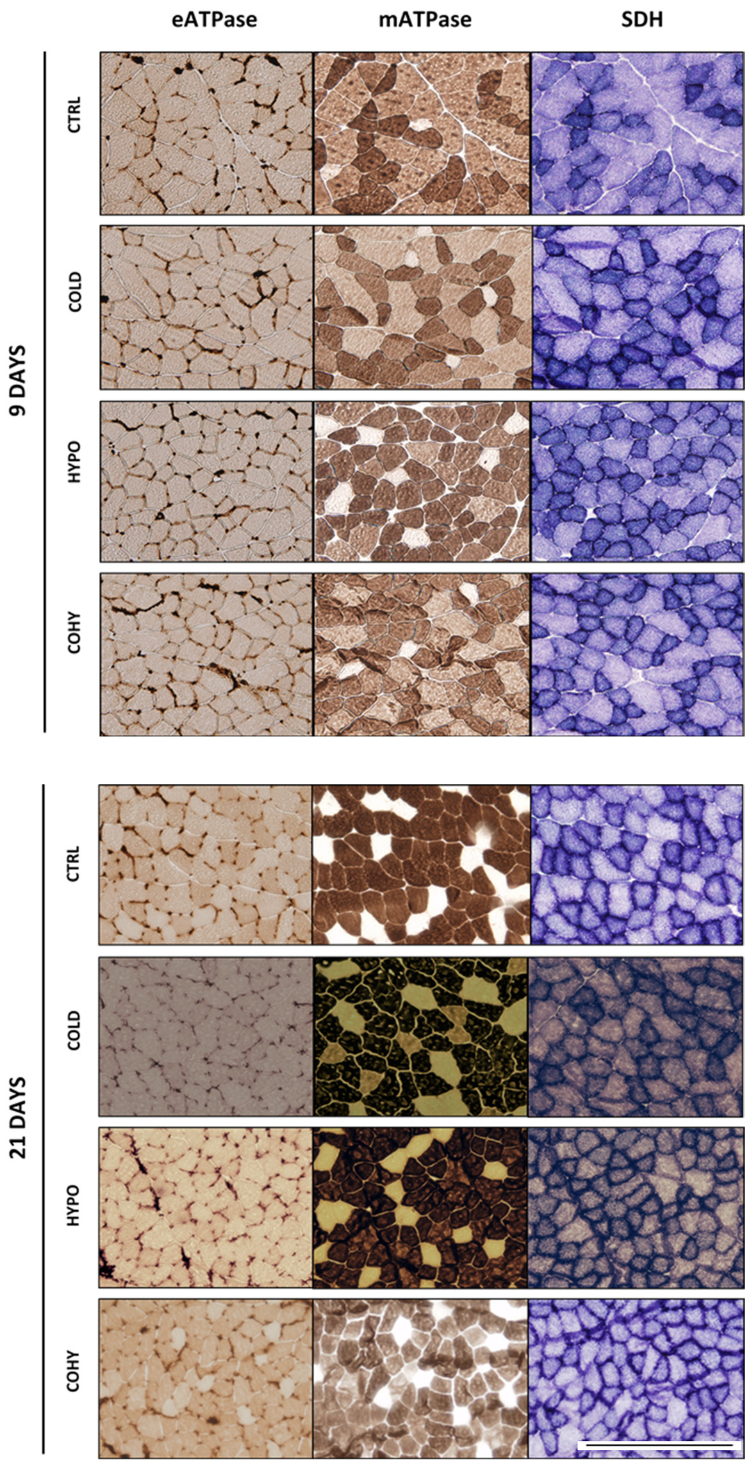

Supplementary Figure 1. Representative microphotographs of gastrocnemius cross-sections from histochemical stains for each experimental group. CTRL, control; COLD, intermittent cold; HYPO, intermittent hypoxia; COHY, intermittent cold + hypoxia; eATPase, endothelial adenosine triphosphatase; mATPase, myofibrillar adenosine triphosphatase after alkaline pre-incubation; SDH, succinate dehydrogenase. All photographs were taken at the same magnification (100x). Bar represents 100 µm.
